# Impact of including weekend days on dietary intake patterns in Korean adults with metabolic syndrome: a secondary data analysis

**DOI:** 10.7586/jkbns.25.066

**Published:** 2026-02-27

**Authors:** Haejung Lee, DaeEun Lee

**Affiliations:** 1 College of Nursing•Research Institute of Nursing Science, Pusan National University, Yangsan, Korea; 2 College of Nursing, Pusan National University, Yangsan, Korea

**Keywords:** Cardiovascular diseases, Diet, Feeding behavior, Metabolic syndrome

## Abstract

**Purpose:**

This study examined the impact of including weekend days in 24-hour dietary recalls on dietary intake patterns and investigated associations between dietary intake—analyzed using nutrient- and food-group–based categories—and cardiovascular risk factors in adults with metabolic syndrome (MetS).

**Methods:**

A cross-sectional study analyzed data from 185 adults aged 40–69 years with metabolic syndrome (MetS), who had either a history of cardiovascular disease or an intermediate-to-high 10-year cardiovascular disease risk. Dietary intake was assessed over three days using 24-hour dietary recalls and analyzed with CAN-Pro 4.0.

**Results:**

Overall dietary intake did not differ significantly according to the number of weekend days included, except food-group–based protein intake (*p* = .030). In the no-weekend group, most nutrients and food groups demonstrated relatively consistent correlations between the three-day average intake and single-day intake (r ≥ .61). In contrast, correlation coefficients varied widely among groups that included weekend days (r = −.08 to .97). Further analyses stratified by specific weekend days (saturdays, sundays, and national holidays) revealed significant differences in nutrient-based protein intake (F = 3.38, *p* = .011). Both nutrient- and food-group–based dietary intakes were significantly associated with cardiovascular risk factors.

**Conclusion:**

The number of weekend days included in dietary assessment may influence estimated dietary intake. The observed variability across specific weekend days suggests that using multiple 24-hour dietary assessments and developing nutritional interventions that explicitly account for weekend schedules would improve the accuracy of dietary pattern assessment and the effectiveness of interventions in individuals with MetS.

## INTRODUCTION

Metabolic syndrome (MetS) is a multifactorial condition defined by the presence of at least three of the following components: abdominal obesity, hypertension, impaired fasting glucose, elevated triglycerides, and reduced high density lipoprotein-cholesterol. The global prevalence of MetS ranges from 12.5% to 31.4%, and its incidence continues to rise [1,2]. In South Korea, the prevalence increased from 21.3% in 2021 to 22.0% in 2022, with notably higher rates observed in older adults—from 17.4%~18.4% in individuals in their 40s to 34.0%~37.9% in those in their 60s [3]. Poor glycemic control and low HDL-C levels among individuals with MetS have been associated with increased all-cause mortality by 19.0% and 11.0%, respectively [4]. Furthermore, the risk of cardiovascular disease (CVD) increases up to 3.92~4.09 times with the accumulation of MetS components [5], highlighting the urgent need for early identification and comprehensive management.

To address MetS-related complications, the Korean Society of Cardiometabolic Syndrome [6] recommends key lifestyle modifications including dietary change, weight management, physical activity, smoking cessation, and alcohol reduction. Among these, dietary modification is particularly effective in improving cardiometabolic outcomes such as systolic blood pressure (SBP), lipid profiles, and body weight [7]. Evidence supports the effectiveness of the Mediterranean diet and Dietary Approaches to Stop Hypertension diets in reducing body mass index (BMI), waist circumference (WC), blood pressure (BP), blood glucose, and lipid levels, with corresponding reductions in CVD risk by 35.0% and 29.0%, respectively [8-10]. Specific food groups also play a protective role: dairy consumption has been associated with a 20.0% lower risk of MetS [11], and higher intakes of vegetables, legumes, and nuts have been linked to 11.0%, 9.0%, and 10.0% reductions in all-cause mortality, respectively [12].

Accurate dietary assessment is essential for effective nutritional intervention and disease management. Common methods include food frequency questionnaires (FFQs), food records, and 24-hour dietary recalls. While FFQs are widely used in epidemiological studies to estimate habitual intake, their limited food lists and fixed portion sizes may compromise accuracy, particularly across diverse populations [13]. In contrast, the 24-hour recall method enables detailed reporting of all foods and beverages consumed in the previous day, with lower respondent burden and no literacy requirement [14]. However, because dietary intake can vary significantly from day to day, single-day recalls may not accurately reflect usual intake. Studies suggest that non-consecutive three-day recalls provide higher accuracy (72.1%) compared to one-day recalls or food records [15], and a minimum of two to three recall days is recommended for reliable estimation [16,17].

Several studies have demonstrated that dietary intake patterns differ significantly between weekdays and weekends [18,19]. For example, diet quality tends to be lower on weekends [18], with notable variations in the types and amounts of foods reported. Energy intake generally increased, whereas the intake of essential nutrients decreased [19]. In addition, Turicchi et al. [20] observed a pattern of weight gain during weekends and weight loss during weekdays, with an overall weight fluctuation of approximately 0.35%. Therefore, it is necessary to examine the impact of weekend inclusion on estimates of energy intake and diet quality when assessing dietary patterns in individuals with MetS.

In individuals with MetS, it is critical to ensure the reliability and consistency of dietary assessment methods. The MyPlate guideline developed by the United States Department of Agriculture (USDA) emphasizes balanced intake across five major food groups—grains, protein foods, fruits, vegetables, and dairy—and has shown positive effects on dietary behaviors and metabolic outcomes [21]. A 12-week MyPlate-based nutrition education program in adults with type 2 diabetes significantly improved blood glucose and hemoglobin A1c (HbA1c) levels [22]. Furthermore, total energy and nutrient intake are closely linked to cardiovascular risk: even modest weight loss of 5.0% through caloric restriction leads to meaningful improvements in BMI, WC, and BP [23], and reduced saturated fat intake significantly lowers total and low-density lipoprotein cholesterol (LDL-C) [24].

Building upon previous findings [5,25], this study aims to explore the influence of weekend day inclusion on dietary intake patterns and to investigate the associations between dietary intake—assessed using both nutrient-based and food-group-based analyses—and cardiovascular risk factors (BMI, WC, BP, blood glucose, HbA1c, and lipid profiles) in adults with MetS.

## METHODS

### 1. Study design

This study employed a cross-sectional design using secondary data derived from a case–control study entitled 'Comparison of health indicators according to the presence or absence of acute coronary syndrome (ACS) in patients with metabolic syndrome.'

### 2. Participants

Participants in the parent study were outpatients aged 40 to 69 years who had been diagnosed with MetS and were receiving treatment at the Endocrinology and Cardiovascular Centers of Pusan National University Hospital, Yangsan, South Korea. Eligibility criteria for the parent study included a documented history of ACS — such as percutaneous coronary intervention or coronary artery bypass grafting— or an intermediate to high 10-year CVD risk, defined by the Predicting Risk of Cardiovascular Disease EVENTs equation score of ≥ 7.5% [26]. Exclusion criteria included (1) participation in other lifestyle intervention trials, (2) clinical instability as determined by the attending physician, and (3) inability to complete questionnaires or communicate effectively. Of the 208 participants enrolled in the parent study, 21 who did not complete the three-day dietary record and 2 who declined blood testing were excluded, leaving 185 in the final analysis. A post hoc power analysis using G*Power (version 3.1.9.7) indicated that, with a total sample size of 185, an α level of .05, and an effect size of f = 0.50 for a one-way analysis of variance with three groups, the achieved power (1–β) was approximately 0.99.

### 3. Instruments

#### 1) General and clinical characteristics

Participants’ demographic and socioeconomic characteristics included in this study were age, sex, marital status, education level, employment status, and monthly income. Health-related behaviors included alcohol consumption, smoking status, engagement in regular physical activity, and overall physical activity levels. Clinical indicators included cardiovascular risk factors such as fasting blood glucose, HbA1c, lipid profiles, BP, BMI, and WC, as well as comorbid conditions such as diabetes, hypertension, and dyslipidemia.

Regular exercise was defined as engaging in physical activity at least three times per week for 20 minutes or more per session over the past month. Physical activity level was evaluated using the Korean short version of the International Physical Activity Questionnaire and categorized as inactive, minimally active, or health-enhancing physical activity [27]. BP, height, weight, WC, and blood tests (fasting blood glucose, HbA1c, and lipid profiles) were measured on the day of the clinic visit. BMI was calculated as weight in kilograms divided by height in meters squared (kg/m^2^) and classified into underweight (< 18.5 kg/m^2^), normal (18.5~24.9 kg/m^2^), and obese (≥ 25.0 kg/m^2^), in accordance with international standards [28]. WC was measured following the guidelines of the Korean Society for the Study of Obesity guideline [29]. WC was measured with a non-stretchable measuring tape at the midpoint between the lower margin of the last palpable rib and the top of the iliac crest with the participant standing [30]. Fasting blood glucose, HbA1c, and lipid profile values were obtained after a minimum of four hours of fasting. HbA1c was measured from 2 mL of whole blood collected in an EDTA tube using an HLC-723 G11 analyzer (Tosoh, Tokyo, Japan). Lipid profiles and fasting glucose levels were analyzed from 2 mL of blood collected in an SST tube using an AU-5800 chemistry analyzer (Beckman Coulter, Brea, USA).

#### 2) Dietary intake assessment

Dietary intake was assessed using 24-hour recalls over three consecutive days (Days 1∼3) following the outpatient visit. If consecutive recording was not feasible, data were collected on three non-consecutive days. Intake data were analyzed using CAN-Pro 4.0 (Computer Aided Nutritional Analysis Program), developed by the Korean Nutrition Society. Both one-day and three-day average intakes were calculated for analysis. Based on previous literature and clinical relevance to MetS, the primary focus was on total energy intake, macronutrients (carbohydrates, protein, fat, saturated fat), sodium, cholesterol, and food group intake (grains, protein foods, vegetables, dairy, and fruits) [6,11,12].

Nutrient intake was evaluated according to the 2020 Dietary Reference Intakes for Koreans [31]. Energy intake was expressed as a percentage of the Estimated Energy Requirement, based on three-day averages. The adequacy of macronutrient intake was assessed by comparing carbohydrate and protein intake with the recommended nutrient intakes, whereas fat and saturated fat intakes were evaluated against the recommended thresholds of 30.0% and 7.0% of total energy intake, respectively. Sodium intake was compared with the Chronic Disease Risk Reduction Intake, and cholesterol intake was evaluated against the 300 mg/day guideline.

Food group intake was analyzed based on the MyPlate dietary guidelines established by the USDA, which recommend balanced consumption across five major food groups—grains, protein foods, vegetables, fruits, and dairy—with standardized serving sizes [21]. Recommended daily intake by energy level was referenced from Dudek [32]. Actual intake rates were calculated by expressing the consumed portions as a percentage of the recommended intake for each food group.

### 4. Data collection

Data collection for the parent study was conducted between April and August 2024. Participants completed structured questionnaires in a private consultation room, followed by anthropometric measurements and blood sample collection. Participants were instructed to complete a food diary for three consecutive days, starting the day after their clinic visit. When completing consecutive three-day records was not feasible, data were collected on three non-consecutive days, as previous studies have shown that the two methods yield comparable accuracy [15]. They recorded all foods and beverages consumed, including portion sizes and preparation methods. Food models and visual aids were provided to assist with accurate portion size estimation. Participants submitted photographs of their completed food diaries via mobile messaging, and follow-up telephone dietary assessments were conducted by research staff to clarify and complete any missing or ambiguous information. To ensure accurate dietary assessment, standardized criteria for estimating dietary intake were established. Data collectors underwent a one-hour training session on dietary assessment procedures and considerations, followed by peer practice sessions prior to data collection. One master’s nursing student and an integrated master’s/PhD student reviewed the completed dietary records. When clarification was needed, they contacted participants by phone to verify or supplement the information.

### 5. Data analysis

All statistical analyses were performed using SPSS version 26.0 (IBM Corp, Armonk, NY, USA). Statistical significance was set at *p* < .05. Participant characteristics were summarized with means and standard deviations for continuous variables and frequencies and percentages for categorical variables. Participants were classified into three groups (0, 1, or 2 weekend days included) based on the three-day dietary recall, in accordance with evidence of weekday–weekend differences in dietary intake [18,19]. Differences in three-day average dietary intake according to the number of weekend days were examined using one-way analysis of covariance (ANCOVA), with BMI and HbA1c as covariates. When the assumption of homogeneity of variance was not met, ranked ANCOVA was performed. Pearson correlation coefficients were calculated to assess the consistency between the three-day average intake and intakes on weekdays, Saturdays, Sundays, and national holidays within each group. For participants without weekend days, consistency was assessed using intakes from first, second, and third days. Differences in three-day average dietary intake according to the inclusion of Saturdays, Sundays, or national holidays were analyzed using ANCOVA, adjusting for BMI and HbA1c. When the assumption of homogeneity of variance was violated, a ranked ANCOVA was applied. Associations between three-day average nutrient and food intakes and CVD risk factors—including BMI, WC, BP, fasting glucose, HbA1c, and lipid profiles—were examined using Pearson correlation coefficients.

### 6. Ethical considerations

This study was exempt from additional ethical review by the Institutional Review Board (IRB) of Pusan National University, Busan, South Korea, in accordance with relevant research ethics guidelines (IRB No. 2025_72_HR). In the parent study, participants were provided with a comprehensive explanation of the study objectives and procedures, and informed consent was obtained prior to data collection. The dataset used in the present study was fully de-identified and contained no personally identifiable information, ensuring that participant privacy and confidentiality were maintained throughout the research process.

## RESULTS

### 1. Participant characteristics and group differences by number of weekend days included

From the original dataset of 208 individuals, 185 participants were included in the final analysis. Twenty-one individuals were excluded due to incomplete three-day dietary recall data, and two were excluded for declining blood testing. These exclusions were made to ensure the completeness and reliability of the data used in this study.

The general characteristics of the study participants are presented in Table 1. A total of 185 individuals were included in the analysis, with a mean age of 60.68 ± 6.13 years; 79.5% (n = 147) were men. Among the participants, 79.5% had spouse, 56.8% had completed high school or less, and 66.5% were employed. Over half (52.4%) reported a monthly income of 4 million KRW or more. Regarding health behaviors, 50.3% were current alcohol consumers, 73.5% were non-smokers, 61.1% engaged in regular physical activity, and 25.9% met criteria for health-enhancing physical activity. The mean BMI was 26.07 ± 3.39 kg/m^2^, and the mean HbA1c level was 6.72 ± 0.91%. The prevalence of comorbidities was as follows: diabetes (75.7%), hypertension (52.4%), and dyslipidemia (54.1%). Among participants with diabetes, nearly half (47.9%) had a disease duration of 10 years or more. Likewise, 48.5% of those with hypertension and 37.0% with dyslipidemia had been diagnosed for at least 10 years.

In terms of nutrient-based intake, the three-day average intake exceeded dietary recommendations for carbohydrates (182.92% ± 55.57%), protein (124.95% ± 46.75%), cholesterol (133.22% ± 69.60%), and sodium (186.30% ± 85.42%). In contrast, total fat (68.29% ± 30.84%) and saturated fat (54.01% ± 42.67%) intakes were below the recommended levels. Regarding food-group-based intake, the three-day average consumption was above the recommended levels for grains (142.29% ± 54.37%) and protein foods (169.67% ± 105.62%), whereas intakes of vegetables (66.13% ± 41.39%), dairy (16.47% ± 20.93%), and fruits (28.53% ± 32.72%) did not meet the recommended levels.

When comparing participant characteristics across groups classified by the number of weekend days included (0, 1, or 2 days), BMI and HbA1c showed statistically significant differences among the groups (*p* < .05). Participants with one weekend day had higher BMI and lower HbA1c levels compared to the other groups. No significant differences were observed for the other variables.

### 2. Comparison of dietary intake by the number of weekend days included

Table 2 presents the differences in dietary intake according to the number of weekend days included (0, 1, or 2). Participants in the no-weekend-day group tended to have higher nutrient-based intakes of total calories, protein, fat, saturated fat, and cholesterol, as well as higher food-group-based intakes of protein, dairy, and fruit. In contrast, participants in the two-weekend-day group tended to have higher nutrient-based intakes of carbohydrates and sodium, as well as higher food-group-based intakes of grains and vegetables. Participants in the one-weekend-day group generally had lower intakes across most dietary components. However, a one-way ANCOVA controlling for BMI and HbA1c indicated that these differences were not statistically significant, except for food-group-based protein intake. The no-weekend-day group exhibited significantly higher protein intake (food-group-based) than other groups (F = 3.59, *p* = .030).

### 3. Consistency between three-day average intake and day-specific intake

Table 3 presents the consistency between three-day average intakes and day-specific intake. In the no-weekend-day group, correlations were relatively stable, ranging from .61 to .91. In the one-weekend-day group, correlations between the three-day average and weekday or Saturday intakes ranged from .65 to .87, whereas correlations with national holiday intakes ranged from −.08 to .87. Notably, the correlations for fat intake (nutrient-based) and protein intake (food group) were particularly low, with r = .02 and r = −.08, respectively. Two participants provided Sunday dietary recall data; therefore, correlations with Sunday intakes were not estimated.

In the two-weekend-day group, correlations between the three-day average and weekday, Saturday, and Sunday intake ranged from .61 to .89, whereas correlations with national holiday intakes ranged from .10 to .90. Within this group, the correlations for fat intake (nutrient-based) and vegetable intake (food group) were particularly low, with r = .10 and r = .20, respectively.

### 4. Dietary intakes on Saturdays, Sundays, and national holidays

Differences in dietary intake on Saturdays, Sundays, and national holidays are presented in Table 4. Participants whose recalls included national holidays generally reported the lowest dietary intakes, whereas those including Sundays showed comparatively higher intakes across most dietary components. A one-way ANCOVA controlling for BMI and HbA1c indicated that, for most nutrients and food groups, there were no statistically significant differences in dietary intake on Saturdays, Sundays, or national holidays, with the exception of protein. Nutrient-based protein intake was significantly lower on national holidays.

### 5. Associations between dietary intake and cardiovascular risk factors

Table 5 summarizes the associations between three-day average dietary intake and cardiovascular risk factors, including fasting glucose, HbA1c, lipid profiles, BP, BMI, and WC. In the nutrient-based analysis, total energy intake was positively associated with BMI (r = .19), WC (r = .17), and triglycerides (r = .20). Carbohydrate intake was negatively correlated with total cholesterol (r = −.15) and LDL-C (r = −.16), whereas protein intake was positively associated with triglycerides (r = .25) and diastolic BP (r = .17). Fat intake showed positive correlations with BMI (r = .15), total cholesterol (r = .15), and triglycerides (r = .29). Saturated fat and cholesterol intakes were negatively associated with fasting glucose, with r = −.15 and r = −.17, respectively.

Regarding food-group-based intake, total energy intake was positively correlated with BMI (r = .16) and triglycerides (r = .20). Protein intake (food-group-based) was positively associated with triglycerides (r = .27), SBP (r = .15), diastolic BP (r = .16), and total cholesterol (r = .16). Vegetable intake was negatively correlated with triglycerides (r = −.15), whereas no significant associations were observed for grains, dairy, or fruit intake.

## DISCUSSION

This study aimed to examine the impact of including weekend days on dietary intake patterns and to explore the associations between dietary intake and cardiovascular risk factors among adults with MetS. Dietary data were collected on three days following outpatient visits. The mean age of participants was 60.68 ± 6.13 years, and the majority were men (79.5%). A substantial proportion of participants had diabetes (75.7%), hypertension (52.4%), and dyslipidemia (54.1%), with a mean BMI of 26.07 ± 3.39 kg/m^2^, indicating an overall trend toward obesity. These characteristics underscore the elevated cardiometabolic risk within this population. Consistent with previous research, the presence of three or more MetS components has been shown to increase the risk of CVD by approximately 3.92 to 4.09 times [5], highlighting the importance of early dietary monitoring and intervention in individuals with MetS.

Despite the elevated cardiometabolic risk profile, 61.1% of participants reported engaging in regular physical activity. However, only 25.9% met the recommended threshold for health-enhancing physical activity. This discrepancy underscores a critical gap between general physical activity and levels sufficient to yield clinically meaningful health benefits. These findings align with those of the Korean Society of Cardiometabolic Syndrome [6], which reported a higher prevalence of MetS among physically inactive individuals. Furthermore, a recent meta-analysis by Alshammary et al. [5] demonstrated that combining aerobic exercise with dietary interventions significantly improves key metabolic indicators—including BMI, WC, BP, lipid profiles, and HbA1c—in individuals with obesity and type 2 diabetes. Taken together, these results emphasize the importance of implementing structured, multifaceted lifestyle modification programs to effectively manage cardiometabolic risk and reduce the burden of chronic disease.

In the present study, no significant differences in dietary intakes were observed according to the number of weekend days included in the dietary assessment, with the exception of food-group-based protein intake. The no-weekend-day group exhibited significantly higher protein intake compared to the other groups, a finding consistent with that of Jahns et al. [33], who reported higher protein intake on weekdays than on weekends. Among working adults, the structured lunch environment on weekdays—such as fixed meal times and access to company cafeterias or workplace food-service facilities—likely increases the probability of consuming a full meal that includes adequate protein. Shin et al. [34] reported that workers who used workplace food-service facilities had significantly higher protein intakes than those who did not. Given that 66.5% of the participants in the present study were employed, it is plausible that weekday dietary patterns were influenced by these structured meal times. Taken together, these findings suggest that the higher dietary intake observed on weekdays may reflect occupational influences on eating behaviors. In addition, Matsumoto et al. [35] reported that higher frequencies of eating out correlated with lower vegetable intake and higher fat consumption. Therefore, assessing eating out patterns may enhance the understanding of participants’ dietary intake.

In contrast, Kant and Graubard [19] found that both men and women had significantly higher total caloric intake and caloric intake from snacks on weekends compared to weekdays, while Bejar [18] found that overall diet quality was 12%⁓26% lower on weekends. These findings differ from those of the present study. Given that 74.3% of our participants had been diagnosed with diabetes for five years or more, 77.4% with hypertension, and 67.0% with dyslipidaemia, it is plausible that they had already implemented dietary and lifestyle modifications in response to their diagnosis. Indeed, Grahovac et al. [36] observed that longer disease duration was significantly associated with greater adherence to the Mediterranean diet. These findings suggest that the weekday–weekend gap in dietary intake, commonly observed in the general population, may have been attenuated in our sample. Moreover, reliance on a three-day dietary recall may have been insufficient to fully capture weekend-related variability in dietary intake. Future research employing longer assessment periods is warranted to clarify whether the inclusion of weekend days influences the accuracy of dietary intake estimates. Additionally, although we compared dietary intake according to the number of weekdays and weekend days included in the assessment, we did not directly analyze individual differences between weekday and weekend dietary patterns or how these differences interact with eating-out behaviors. Therefore, future studies should examine the interplay between weekday–weekend dietary variation and eating-out patterns in greater depth.

Consistency between three-day average intakes and day-specific intakes showed substantial variation in the weekend-included groups, whereas the no-weekend-day group demonstrated relatively stable intakes. In the one-weekend-day group, national-holiday intakes showed weak correlations with three-day average dietary intakes. Specific weak correlations were observed for nutrient-based intakes of protein, fat, and cholesterol, as well as food-group-based intakes of protein and dairy. In the two-weekend-day group, national holiday intakes demonstrated very low correlations with nutrient-based intakes of fat and sodium and food-group-based vegetable intakes. However, sample size for national holidays was particularly small and the absence of within-subject comparisons across Saturdays, Sundays, and national holiday limits the interpretability of these findings. Future research should examine intra-individual variability in weekend and national holiday dietary intake and include more than 50 participants to obtain more reliable correlation estimates [37].

When dietary variation was compared according to the weekend-day type, significant differences in protein intake were observed among Saturdays, Sundays, and national holidays, with the lowest intake occurring on national holidays. An et al. [38] reported the highest intakes of total energy, fat, saturated fat, and sodium on Saturdays among U.S. adults, whereas Gingrich et al. [39] found that protein intake was significantly higher on Sundays in older German adults. Although our findings exhibit a similar pattern, direct comparisons should be interpreted with caution due to differences in ethnicity, employment status, and the prevalence of chronic diseases across study populations.

In this study, dietary intake was significantly associated with cardiovascular risk factors. At the nutrient level, total energy intake showed significant positive correlations with BMI, WC, and triglyceride levels, highlighting the impact of excessive energy consumption on obesity. Fat intake was positively associated with BMI and triglyceride levels, while protein intake was positively associated with triglyceride levels. In contrast, when dietary intake was examined by food groups, consumption of protein-rich foods was positively associated with BP, total cholesterol, and triglyceride levels, suggesting a potential contribution to lipid dysregulation as well as a limitation of the food group–based assessment. Specifically, food group assessment does not distinguish between high-fat protein sources (e.g., red meat, processed meat) and lean protein sources (e.g., legumes, poultry, fish). In our study, protein intake exceeded dietary recommendations, both at the nutrient level and according to food-group-based assessments. Consistent with previous research [25], plant-based protein has been linked to beneficial effects such as reduced SBP, whereas animal protein intake is associated with adverse metabolic outcomes. Because food group-based assessment does not differentiate between plant and animal protein sources, refining the classification of protein foods to account for fat content—particularly saturated fat—could enhance the accuracy of dietary analysis and better reflect the physiological role of high-quality protein in human health.

Interestingly, in contrast to previous evidence, intake of carbohydrates, saturated fat, and cholesterol in this study was negatively correlated with glucose, total cholesterol, and LDL-C levels. One plausible explanation is reverse causality: participants with elevated metabolic markers may have already begun dietary modifications—such as reducing carbohydrate, saturated fat, and cholesterol intake—following clinical diagnosis. However, these recent dietary changes may not yet have produced measurable improvements in blood biomarkers. Additionally, the use of a short-term dietary assessment (three-day recall) may limit the ability to accurately capture habitual intake, particularly for nutrients with cumulative or delayed metabolic effects. Future research using longitudinal data is warranted to confirm these associations and to better control for confounding factors such as medication use, disease status, and total energy intake.

In this study, vegetable intake was inversely associated with triglyceride levels, reinforcing its known benefits for lipid metabolism. Although fruit and dairy intake were not significantly associated with CVD risk factors in this study, previous research [12,33,36] has consistently demonstrated their protective effects. Higher consumption of fruits and dairy products has been linked to a reduced risk of MetS and cardiovascular mortality. However, in this population, reported intakes of vegetables, fruits, and dairy products fell below recommended dietary guidelines. Evidence from prior studies suggests that MyPlate-based interventions can effectively improve both glycemic control and overall dietary quality [22], supporting the practical utility of the MyPlate guidelines, which promote balanced eating by visually illustrating appropriate portion sizes and distribution across food groups. Taken together, these findings underscore the importance of improving adherence to dietary guidelines—particularly for fruits, vegetables, and dairy products—as part of a comprehensive strategy to mitigate cardiometabolic risk. Such approaches may enhance the effectiveness of dietary interventions and inform public health efforts targeting populations at elevated risk.

This study aimed to explore the impact of the number of weekend days included in 24-hour dietary recalls among adults with MetS, thereby evaluating the reliability and representativeness of this dietary assessment method. Furthermore, by analyzing the associations between dietary intake and cardiovascular risk factors in individuals with MetS, the study contributes to the validation of dietary assessment methods and demonstrates their potential clinical applicability. Nonetheless, several limitations should be acknowledged. First, this study is based on cross-sectional data, which inherently limits the ability to establish causal relationships among variables. Second, the sample was restricted to Korean adults aged 40~69 with metabolic syndrome, thereby limiting the generalizability of the findings to other ethnicities or age groups. Third, intra-individual comparisons between weekday and weekend dietary intake were not possible, restricting the assessment of within-person variation. Fourth, the sample size was insufficient to robustly analyze correlations between the three-day average intake and national holiday consumption in the weekend-included groups. Additionally, correlations between the three-day average intake and Sunday intakes in the one-weekend-day group were not estimated due to the very small sample size (n = 2). Purposeful sampling of weekend and national holiday dietary intakes could enhance the validity of the observed dietary variation. Finally, due to the use of secondary data, detailed information on weekend and weekday dietary patterns-such as meal frequency, location, or type of meal-could not be examined.

## CONCLUSION

This study demonstrates that the number of weekend days included in dietary assessments did not significantly affect overall intake in this sample. However, variation among specific weekend days suggests that potential day-to-day fluctuations should be taken into account. Future dietary assessments should capture both weekend and weekday patterns and consider contextual behaviors, such as meal frequency, dining out, and meal location, in order to enable more precise dietary analysis and tailored nutritional education. Based on these findings, we propose the following recommendations: (1) conduct studies comparing weekday versus weekend intake within the same individuals, purposefully including comparable numbers of weekdays, weekends, and national holidays; (2) incorporate meal frequency, context, and location into dietary surveys to better inform intervention programs; and (3) examine whether consecutive versus non-consecutive dietary recording days influence intake estimates within participants.

## Figures and Tables

**Table 1.** General Characteristics of the Participants (N = 185)

Variables	Categories	Total (n = 185)	No-weekend-day (n = 62)	One-weekend-day (n = 56)	Two-weekend-day (n = 67)	*χ*^2^/F (*p*)
Age (years)	40∼49	15 (8.1)	6 (9.7)	5 (8.9)	4 (6.0)	1.42 (.841)
	50∼59	50 (27.0)	15 (24.2)	14 (25.0)	21 (31.3)	
	60∼69	120 (64.9)	41 (66.1)	37 (66.1)	42 (62.7)	
		60.68 ± 6.13	60.90 ± 6.11	60.00 ± 6.36	61.03 ± 6.01	0.49 (.612)
Sex	Men	147 (79.5)	45 (72.6)	44 (78.6)	58 (86.6)	3.90 (.142)
	Women	38 (20.5)	17 (27.4)	12 (21.4)	9 (13.4)	
Spouse	Yes	147 (79.5)	49 (79.0)	41 (73.2)	57 (85.1)	2.64 (.267)
	No	38 (20.5)	13 (21.0)	15 (26.8)	10 (14.9)	
Education	≤ High school	105 (56.8)	33 (53.2)	33 (58.9)	39 (58.2)	0.48 (.787)
	≥ University	80 (43.2)	29 (46.8)	23 (41.1)	28 (41.8)	
Employed	Yes	123 (66.5)	34 (54.8)	41 (73.2)	48 (71.6)	5.71 (.058)
	No	62 (33.5)	28 (45.2)	15 (26.8)	19 (28.4)	
Total monthly income (10,000 won)	< 200	41 (22.2)	15 (24.2)	12 (21.4)	14 (20.9)	1.72 (.788)
	200∼399	47 (25.4)	17 (27.4)	16 (28.6)	14 (20.9)	
	≥ 400	97 (52.4)	30 (48.4)	28 (50.0)	39 (58.2)	
Drinking	Yes	93 (50.3)	29 (46.8)	31 (55.4)	33 (49.3)	0.91 (.634)
	No	92 (49.7)	33 (53.2)	25 (44.6)	34 (50.7)	
Smoking	Yes	49 (26.5)	18 (29.0)	13 (23.2)	18 (26.9)	0.52 (.771)
	No	136 (73.5)	44 (71.0)	43 (76.8)	49 (73.1)	
Regular exercise	Yes	113 (61.1)	37 (59.7)	34 (60.7)	42 (62.7)	0.13 (.938)
	No	72 (38.9)	25 (40.3)	22 (39.3)	25 (37.3)	
Physical activity	Inactive	58 (31.4)	20 (32.3)	19 (33.9)	19 (28.4)	0.72 (.949)
	Minimally active	79 (42.7)	25 (40.3)	23 (41.1)	31 (46.3)	
	HEPA	48 (25.9)	17 (27.4)	14 (25.0)	17 (25.4)	
Body mass index (kg/m^2^)	< 18.5	1 (0.5)	1 (1.6)	-	-	8.50 (.075)
	18.5∼< 25.0	72 (39.0)	24 (38.7)	15 (26.8)	33 (49.3)	
	≥ 25.0	112 (60.5)	37 (59.7)	41 (73.2)	34 (50.7)	
		26.07 ± 3.39	25.96 ± 3.84	26.96 ± 3.11	25.44 ± 3.05	3.22 (.042)
HbA1c (%)	< 6.9	107 (57.8)	32 (51.6)	39 (69.6)	38 (56.7)	4.16 (.125)
	≥ 6.9	78 (42.2)	30 (48.4)	17 (30.4)	29 (43.3)	
		6.72 ± 0.91	6.86 ± 0.91	6.55 ± 0.83	6.73 ± 0.95	1.77 (.042)
Glucose (mg/dL)		143.51 ± 53.22	144.11 ± 54.44	145.21 ± 59.01	141.52 ± 47.41	0.08 (.924)
CVD risk factors						
Diabetes mellitus	No	45 (24.3)	14 (22.6)	15 (26.8)	16 (23.9)	0.29 (.863)
	Yes	140 (75.7)	48 (77.4)	41 (73.2)	51 (76.1)	
Diagnosis duration (years)	< 5	36 (25.7)	9 (18.8)	14 (34.1)	13 (25.5)	9.56 (.049)
	5~9	37 (26.4)	8 (16.7)	11 (26.8)	18 (35.3)	
	≥ 10	67 (47.9)	31 (64.5)	16 (39.1)	20 (39.2)	
Hypertension	No	88 (47.6)	27 (43.5)	24 (42.9)	37 (55.2)	2.47 (.290)
	Yes	97 (52.4)	35 (56.5)	32 (57.1)	30 (44.8)	
Diagnosis duration (years)	< 5	22 (22.6)	8 (22.9)	9 (28.1)	5 (16.7)	5.45 (.245)
	5~9	28 (28.9)	6 (17.1)	10 (31.3)	12 (40.0)	
	≥ 10	47 (48.5)	21 (60.0)	13 (40.6)	13 (43.3)	
Hyperlipidemia	No	85 (45.9)	36 (58.1)	22 (39.3)	27 (40.3)	5.53 (.063)
	Yes	100 (54.1)	26 (41.9)	34 (60.7)	40 (59.7)	
Diagnosis duration (years)	< 5	33 (33.0)	4 (15.4)	16 (47.0)	13 (32.5)	7.77 (.100)
	5~9	30 (30.0)	8 (30.8)	9 (26.5)	13 (32.5)	
	≥ 10	37 (37.0)	14 (53.8)	9 (26.5)	14 (35.0)	
Nutrients (%)^†^	Total calorie	81.87 ± 24.57	84.88 ± 27.38	81.37 ± 20.94	79.50 ± 24.71	0.79 (.458)
	CHO	182.92 ±55.57	181.60 ± 65.01	183.24 ± 49.59	183.87 ± 51.49	0.03 (.972)
	Proteins	124.95 ±46.75	133.65 ± 48.71	120.55 ± 43.04	120.58 ± 47.42	1.63 (.200)
	Fat	68.29 ± 30.84	73.95 ± 33.24	65.86 ± 27.79	65.05 ± 30.68	1.60 (.205)
	Saturated fat	54.01 ± 42.67	59.22 ± 49.42	48.38 ± 31.16	53.90 ± 44.24	0.95 (.389)
	Sodium	186.30 ± 85.42	188.93 ± 98.61	172.47 ± 62.82	195.43 ± 88.38	1.15 (.319)
	Cholesterol	133.22 ± 69.60	146.70 ± 71.47	119.91 ± 66.15	131.86 ± 69.40	2.23 (.111)
Food groups (%)^‡^	Grains	142.29 ± 54.37	140.25 ± 57.12	143.42 ± 48.97	143.24 ± 56.74	0.07 (.937)
	Proteins	169.67 ± 105.62	188.57 ± 112.78	165.73 ± 93.80	155.48 ± 107.03	1.65 (.195)
	Vegetables	66.13 ± 41.39	64.19 ± 43.91	63.85 ± 32.66	69.84 ± 45.63	0.42 (.657)
	Dairy	16.47 ± 20.93	18.47 ± 22.41	14.40 ± 19.92	16.34 ± 20.47	0.56 (.574)
	Fruits	28.53 ± 32.72	28.78 ± 31.25	26.60 ± 31.65	29.93 ± 35.25	0.16 (.853)

Values are presented as the mean ± standard deviation or n (%). HEPA = Health-enhancing physical activities; HbA1c = Hemoglobin A1c; CVD = Cardiovascular disease; CHO = Carbohydrate.

† Percentage of actual three-day average intake ratio to recommended nutrient intake (1. Total calorie: men = [40 ≤ age < 50: 2,500 kcal, 50 ≤ age < 65: 2,200 kcal, 65 ≥ age: 2,000 kcal], women = [40 ≤ age < 50: 1,900 kcal, 50 ≤ age < 65: 1,700 kcal, 65 ≥ age: 1,600 kcal], 2. CHO: 130 g, 3. Protein: men = [4 0≤ age < 50: 65 g, 50 ≥ age: 60 g], women = 50g, 4. Acceptable macronutrient distribution range [fat intake: 30% of total energy; saturated fat: < 7.0% of total energy], 5. Chronic disease risk reduction intake [sodium: age < 65 yr: 2300 mg, ≥ 65 yr: 2100 mg], 6. Cholesterol: less than 300 mg/day);

‡ Percentage of actual three-day average intake/recommended foods intake in the MyPlate guideline.

**Table 2.** Comparison of Dietary Intake by the Number of Weekend Days Included (N = 185)

Variables		Three-day average (n = 185)	Three-day average			F	*p*
			No-weekend-day (n = 62)	One-weekend-day (n = 56)	Two-weekend-day (n = 67)		
Nutrients	Total calorie (kcal)	1686.87 ± 523.73	1739.21 ± 628.25	1677.28 ± 418.22	1646.03 ± 499.96	0.13^‡^	.881
	CHO (g)	237.72 ± 72.37	236.07 ± 84.51	238.21 ± 64.47	239.03 ± 66.94	0.20^‡^	.822
	Proteins (g)	72.97 ± 27.78	77.67 ± 30.39	70.20 ± 24.88	70.73 ± 27.62	1.24^‡^	.291
	Fat (g)	47.08 ± 22.18	51.07 ± 26.13	45.16 ± 17.93	44.96 ± 21.20	0.91^‡^	.403
	Saturated fat (g)	8.66 ± 6.72	9.37 ± 7.55	7.83 ± 5.15	8.71 ± 7.06	0.45^‡^	.641
	Sodium (mg)	4164.83 ± 1874.45	4227.88 ± 2189.24	3878.18 ± 1409.20	4344.16 ± 1900.61	1.04^‡^	.355
	Cholesterol (mg)	399.99 ± 208.63	440.09 ± 214.42	359.74 ± 198.47	395.59 ± 208.19	2.30^†^	.061
Food groups	Total calorie (kcal)	1497.07 ± 442.90	1497.07 ± 520.56	1428.92 ± 377.53	1432.20 ± 418.71	0.06^‡^	.938
	Grains (g)	274.03 ± 102.83	268.00 ± 116.18	268.70 ± 87.70	284.18 ± 102.10	0.73^‡^	.485
	Proteins (g)	251.95 ± 127.74	284.56 ± 136.65	243.55 ± 122.06	228.73 ± 119.25	3.59^‡^	.030
	Vegetables (g)	338.81 ± 188.22	313.06 ± 182.16	323.18 ± 180.42	375.68 ± 196.91	2.71^‡^	.069
	Dairy (g)	66.74 ± 103.72	76.22 ± 119.18	62.51 ± 104.60	61.51 ± 87.30	0.61^‡^	.544
	Fruits (g)	141.36 ± 161.09	149.00 ± 170.68	126.52 ± 142.30	146.70 ± 168.16	0.57^‡^	.568

Values are presented as the mean ± standard deviation. CHO = Carbohydrate.

† One-way analysis of covariance adjusted for body mass index and hemoglobin A1c;

‡ Ranked one-way analysis of covariance adjusted for body mass index and HbA1c.

**Table 3.** Consistency between Three-day Average Intakes and Day-specific Intakes According to Weekend Inclusion Group (N = 185)

Variables		No weekend days (n = 62)			One weekend day (n = 56)			Two weekend days (n = 67)			
		Three-day avg. vs. 1st day (n = 62)	Three-day avg. vs. 2nd day (n = 62)	Three-day avg. vs. 3rd day (n = 62)	Three-day avg. vs. weekdays (n = 56)	Three-day avg. vs. Saturday (n = 38)	Three-day avg. vs. national holiday (n = 16)	Three-day avg. vs. weekday (n = 67)	Three-day avg. vs. Saturday (n = 67)	Three-day avg. vs. Sunday (n = 62)	Three-day avg. vs. national holiday (n = 5)
		r (*p*)									
Nutrients	Total calorie (kcal)	.78 (<.001)	.88 (<.001)	.85 (<.001)	.92 (<.001)	.70 (<.001)	.54 (.030)	.81 (<.001)	.79 (<.001)	.86 (<.001)	.60 (.285)
	CHO (g)	.86 (<.001)	.90 (<.001)	.91 (<.001)	.92 (<.001)	.87 (<.001)	.75 (<.001)	.73 (<.001)	.84 (<.001)	.84 (<.001)	.80 (.044)
	Proteins (g)	.68 (<.001)	.82 (<.001)	.77 (<.001)	.91 (<.001)	.66 (<.001)	.44 (.085)	.82 (<.001)	.80 (<.001)	.82 (<.001)	.90 (.037)
	Fat (g)	.66 (<.001)	.80 (<.001)	.65 (<.001)	.92 (<.001)	.69 (<.001)	.02 (.948)	.80 (<.001)	.66 (<.001)	.74 (<.001)	.10 (.873)
	Saturated fat (g)	.77 (<.001)	.73 (<.001)	.61 (<.001)	.77 (<.001)	.77 (<.001)	.57 (.022)	.79 (<.001)	.61 (<.001)	.68 (<.001)	.60 (.285)
	Sodium (mg)	.80 (<.001)	.89 (<.001)	.80 (<.001)	.89 (<.001)	.81 (<.001)	.87 (<.001)	.80 (<.001)	.76 (<.001)	.82 (<.001)	.20 (.747)
	Cholesterol (mg)	.61 (<.001)	.74 (<.001)	.67 (<.001)	.91 (<.001)	.71 (<.001)	.47 (.069)	.76 (<.001)	.72 (<.001)	.64 (<.001)	.65 (.231)
Food groups	Total calorie (kcal)	.75 (<.001)	.86 (<.001)	.83 (<.001)	.94 (<.001)	.72 (<.001)	.62 (.010)	.83 (<.001)	.77 (<.001)	.85 (<.001)	.70 (.188)
	Grains (g)	.78 (<.001)	.88 (<.001)	.84 (<.001)	.93 (<.001)	.69 (<.001)	.59 (.015)	.73 (<.001)	.68 (<.001)	.72 (<.001)	.60 (.285)
	Proteins (g)	.70 (<.001)	.68 (<.001)	.67 (<.001)	.93 (<.001)	.65 (<.001)	−.08 (.778)	.77 (<.001)	.63 (<.001)	.80 (<.001)	.80 (.104)
	Vegetables (g)	.80 (<.001)	.84 (<.001)	.89 (<.001)	.94 (<.001)	.74 (<.001)	.75 (<.001)	.72 (<.001)	.89 (<.001)	.83 (<.001)	.20 (.747)
	Dairy (g)	.75 (<.001)	.75 (<.001)	.78 (<.001)	.97 (<.001)	.86 (<.001)	.41 (.117)	.72 (<.001)	.80 (<.001)	.74 (<.001)	.46 (.437)
	Fruits (g)	.80 (<.001)	.77 (<.001)	.77 (<.001)	.95 (<.001)	.86 (<.001)	.84 (<.001)	.79 (<.001)	.86 (<.001)	.86 (<.001)	.41 (.493)

Avg. = Average; CHO = Carbohydrate.

**Table 4.** Dietary Intake on Saturday, Sunday, and National Holidays (190 Dietary Intake Records from 123 Participants) (N = 123)

Variables	Saturday (n = 105)	Sunday (n = 64)	National holiday (n = 21)	F	*p*
Nutrients					
Total calorie (kcal)	1664.79 ± 555.16	1681.92 ± 596.48	1462.79 ± 371.84	1.67^†^	.160
CHO (g)	243.85 ± 84.78	236.93 ± 80.11	231.62 ± 52.89	0.05^‡^	.954
Proteins (g)	68.85 ± 28.58	75.22 ± 32.85	53.29 ± 21.05	3.38^†^	.011
Fat (g)	44.36 ± 24.14	47.02 ± 30.94	35.52 ± 20.35	1.03^‡^	.361
Saturated fat (g)	8.48 ± 8.73	10.21 ± 14.96	5.84 ± 6.78	0.78^‡^	.458
Sodium (mg)	4274.64 ± 2486.71	4395.13 ± 2241.95	3937.83 ± 1829.57	0.68^‡^	.508
Cholesterol (mg)	398.22 ± 278.15	377.83 ± 253.32	255.47 ± 243.49	1.99^‡^	.140
Food groups					
Total calorie (kcal)	1432.34 ± 452.37	1465.32 ± 473.56	1270.36 ± 369.31	1.19^‡^	.306
Grains (g)	281.90 ± 134.47	275.72 ± 147.33	272.83 ± 81.12	0.35^‡^	.703
Proteins (g)	222.15 ± 135.44	259.89 ± 170.99	153.99 ± 110.42	2.73^‡^	.068
Vegetables (g)	357.84 ± 228.98	378.46 ± 259.77	356.16 ± 215.04	0.07^‡^	.932
Dairy (g)	62.87 ± 114.17	59.44 ± 121.12	38.17 ± 80.40	0.16^‡^	.851
Fruits (g)	156.04 ± 208.94	139.36 ± 179.59	145.71 ± 164.90	0.03^‡^	.969

Values are presented as the mean ± standard deviation. CHO = Carbohydrate.

^†^One-way analysis of covariance adjusted for body mass index and hemoglobin A1c; ^‡^Ranked one-way analysis of covariance adjusted for body mass index and hemoglobin A1c.

**Table 5.** Associations of Dietary Intake Estimated by Nutrients and Food Group with Cardiovascular Disease Risk Factors (N = 185)

Variables	Three-day average												
	Nutrients							Food groups					
	TCal	CHO	Protein	Fat	SFA	Sodium	Chol	TCal	Grains	Protein	Veg	Dairy	Fruits
	r (*p*)							r (*p*)					
BMI (kg/m^2^)	.19 (.009)	.11 (.143)	.14 (.061)	.15 (.037)	−.03 (.647)	.09 (.204)	.04 (.596)	.16 (.026)	.06 (.391)	.14 (.061)	.08 (.305)	−.03 (.709)	.09 (.246)
WC (cm)	.17 (.023)	.09 (.245)	.11 (.128)	.10 (.194)	−.01 (.890)	.09 (.212)	.01 (.950)	.13 (.086)	.06 (.421)	.13 (.080)	.05 (.538)	−.05 (.501)	.05 (.530)
SBP (mmHg)	.05 (.522)	−.04 (.582)	.10 (.189)	.06 (.417)	.07 (.360)	.06 (.404)	.05 (.481)	.02 (.755)	−.01 (.875)	.15 (.049)	.03 (.653)	.03 (.642)	−.11 (.154)
DBP (mmHg)	.13 (.077)	.01 (.935)	.17 (.018)	.12 (.107)	.06 (.447)	.13 (.073)	.07 (.376)	.08 (.270)	−.01 (.866)	.16 (.026)	.02 (.829)	−.01 (.866)	−.09 (.213)
Glucose (mg/dL)	−.09 (.247)	−.07 (.367)	−.09 (.178)	−.06 (.448)	−.15 (.037)	−.01 (.911)	−.17 (.019)	−.07 (.320)	−.06 (.437)	−.10 (.172)	−.03 (.686)	−.04 (.555)	−.08 (.275)
HbA1c (%)	−.11 (.126)	−.11 (.149)	−.05 (.476)	−.08 (.291)	−.04 (.597)	−.11 (.156)	−.12 (.099)	−.08 (.253)	−.12 (.103)	−.00 (.974)	.05 (.492)	−.24 (.745)	.01 (.945)
TC (mg/dL)	.01 (.891)	−.15 (.037)	.11 (.128)	.15 (.036)	.07 (.379)	.02 (.816)	.05 (.539)	.01 (.957)	−.11 (.138)	.16 (.025)	.01 (.939)	−.06 (.457)	−.03 (.642)
HDL-C (mg/dL)	−.07 (.369)	−.11 (.129)	−.04 (.592)	−.04 (.600)	−.07 (.352)	.06 (.458)	.00 (.972)	−.09 (.208)	−.10 (.157)	−.01 (.889)	.15 (.050)	−.14 (.054)	−.07 (.315)
LDL-C (mg/dL)	−.10 (.157)	−.16 (.029)	−.02 (.695)	−.01 (.961)	.04 (.594)	−.05 (.471)	−.03 (.687)	−.10 (.167)	−.14 (.053)	.02 (.832)	.04 (.574)	−.01 (.867)	−.01 (.992)
TG (mg/dL)	.20 (.005)	.02 (.728)	.25 (<.001)	.29 (<.001)	.10 (.190)	.07 (.343)	.12 (.119)	.20 (.006)	.08 (.289)	.27 (<.001)	−.15 (.046)	−.05 (.523)	−.03 (.732)

TCal = Total calories; CHO = Carbohydrate; SFA = Saturated fatty acid; Chol = Cholesterol; Veg = Vegetable; BMI = Body mass index; WC = Waist circumference; SBP = Systolic blood pressure; DBP = Diastolic blood pressure; HbA1c = Hemoglobin A1c; TC = Total cholesterol; HDL-C = High-density lipoprotein cholesterol; LDL-C = Low-density lipoprotein cholesterol; TG = Triglyceride.

## Data Availability

Data available on request due to privacy/ethical restrictions.
